# Assessment of Knowledge Levels Following an Education Program for Parents of Children With Inflammatory Bowel Disease

**DOI:** 10.3389/fped.2020.00475

**Published:** 2020-08-12

**Authors:** Angharad Vernon-Roberts, Richard B. Gearry, Andrew S. Day

**Affiliations:** ^1^Department of Paediatrics, University of Otago, Christchurch, New Zealand; ^2^Department of Medicine, University of Otago, Christchurch, New Zealand

**Keywords:** residential camp, educational intervention, internal consistency, IBD-KID2, knowledge deficiencies

## Abstract

Children with inflammatory bowel disease (IBD) and their parents have increasing roles in disease management and require sufficient, appropriate information for communication with their clinical team. Formal education is effective at improving disease knowledge, disease outcomes, and mental health, yet few interventions have been targeted for parents of children with IBD. A two day parent education program was held at the annual residential camp for children with IBD in New Zealand with knowledge levels tested pre and post intervention using a validated assessment tool: IBD-KID2. Thirty parents consented, 25 completed the study, 70% were female and 83% had a child with Crohn's disease. The pre-intervention mean score (maximum fifteen) was 10.6 (*SD* 2.9), with no associations with independent variables. Knowledge levels increased significantly following the education program to a mean 12.6 (*SD* 2.0) (*p* < 0.005). Disease specific knowledge may improve outcomes and should, therefore, be reinforced during clinic encounters, and regarded as an ongoing endeavor.

## Introduction

Parents of children with chronic illness have long expressed a need for greater information regarding their child's condition (1). Parents and their children with inflammatory bowel disease (IBD) are now having increased roles in their disease management and decision-making, and crucial to this process is the effective exchange of knowledge and information between the clinical team and the family (1). Targeted programs for families of children with IBD remain scarce, despite their proven efficacy in other chronic disease groups at improving knowledge and disease outcomes, mental health, communication, and problem-solving skills (2–4). Education programs have been designed specifically for families of adults with inflammatory bowel disease (IBD), showing significant improvements in knowledge levels (5, 6).

Identifying opportunities for delivering formal education may be challenging for parents, therefore, utilizing situations where their children are already involved may be beneficial and logistically appealing. Residential camps for children with chronic conditions, including IBD, have been run for many years (7) and have shown a number of benefits for children such as confidence, acceptance, perspective about disease trajectories, and peer connections (8–10). They also consistently demonstrate positive and sustained improvements to knowledge, self-management skills and health related quality of life for children with IBD (8, 11–13). The annual residential camp for children with IBD in New Zealand (Camp Purple, in association with the Crohn's and Colitis New Zealand) includes a two day parent education seminar. Camp Purple provides a convenient and practical setting for the provision of parent education, although no studies have been identified that measured the effect of such a uniquely placed intervention on parent knowledge levels.

### Aims

The objective of this study was to test the disease and treatment knowledge levels of parents before, and after, attending a two-day education program. The overall aim was to assess the effect of the education program on the knowledge levels of parents using a validated knowledge assessment tool: IBD-KID2 (14), and to establish whether IBD-KID2 has the sensitivity to detect change pre and post intervention.

## Methodology

### Population and Ethics

The study was undertaken during Camp Purple in January 2019 in Christchurch, New Zealand. All parents attending the Camp Purple parent education program were invited to participate in the research. Ethical approval for the study was provided by the University of Otago Human Ethics Committee (Health) (H16/116). Written informed consent was obtained from all participants.

### Education Program

The education program was delivered over 2 days and sessions were presented by experts in the field from within New Zealand.

Sessions delivered on day one included an IBD overview, surgical aspects, adolescent health, coping with chronic illness, Crohn's and Colitis New Zealand support group, question and answer session. Sessions delivered on day two were focused on old and new IBD therapies, the nursing role in management of IBD, nutrition and IBD, the psychosocial impact of IBD, epidemiology of IBD, discussions, and questions (Supplemental Digital Content 1).

### Outcome Measure

Knowledge levels were assessed using IBD-KID2, which is a 15 items knowledge assessment tool comprised of six multiple choice questions and nine true/false questions. Participant responses are scored as one for each correct answer, to a maximum total of fifteen. Knowledge domains include general IBD, treatment, and lifestyle/nutrition (Supplemental Digital Content 2). The original IBD-KID2 development study showed good validity and reliability for the assessment of knowledge in children with IBD when compared with a number of comparator groups (14). Further work has established that it has validity, reliability, and generalizability for children with IBD and their parents (manuscript in preparation).

IBD-KID2 was completed at two time points; the initial completion was in paper form before the education program began, the second was an online version emailed 14 days later using a secure, HIPAA compliant form provider.

### Statistical Analysis

Baseline data were collected to provide comparisons of mean scores against demographic and disease specific independent variables. The comparison of mean scores for categorical variables was performed using independent *t*-tests, and ANOVA with Tukey's *post-hoc* analysis. For continuous variables, significance was tested using linear regression. The significance level was considered as *p* ≤ 0.05. Knowledge levels were examined for each IBD-KID2 item to ascertain areas of good and poor knowledge. Poor knowledge was considered when <50% of participants answered an item correctly.

For longitudinal data the mean scores for the baseline and repeat administrations were compared using paired sample *t*-tests. Consistency was examined using the intraclass correlation coefficient (ICC), with a value over 0.8 showing good reproducibility. Change in percentage scores for items were examined between the two time points, in parallel with the percentage of “don't know” answers given.

## Results

### Participants

Thirty-six parents attended the education program at Camp Purple. Thirty consented to the study and completed IBD-KID2 at baseline, with 25 also completing the repeat administration. The mean age of participants was 41 years (*SD* 7.2), 21 (70%) were female. Twenty-five (83%) had children with Crohn's disease (CD), five (17%) ulcerative colitis, and the mean time since diagnosis was 3.9 years (*SD* 3.3). Thirteen (43%) of participants had attended Camp Purple education sessions in previous years.

### IBD-KID2 Scores at Baseline

The mean score for IBD-KID2 at baseline for the group overall was 10.6 (*SD* 2.9) out of a maximum of 15 points. Scores were not significantly associated with any independent linear variables (Table 1). Further analysis of parent education level data showed that mean IBD-KID2 scores were lower for parents with a High School education level (9.2, *SD* 3.7) than those with post-secondary education (11.24, *SD* 2.3) but this did not reach significance (*p* 0.08).

**Table 1:** Mean IBD-KID2 scores: association with independent variables.

**Linear variables**	**Regression (*R*)**	***P*-value**	
Parent age	0.024	0.898	
Child's age	0.319	0.085	
Child's age at diagnosis	0.249	0.185	
Time since diagnosis	0.079	0.678	
**Categorical variables**	**Group**	**Mean IBD-KID2** **score (*****SD*****)**	***P*****-value**
Parent gender	Male	9.8 (2.9)	0.297
	Female	11 (2.9)	
Child's diagnosis	CD	10.8 (3.1)	0.601
	UC	10 (1.4)	
Parent level of education	High school	9.2 (3.7)	0.283
	College	10.7 (2.4)	
	University	11.4 (2.5)	
	Post-graduate	12.3 (1.2)	
Family ethnicity	NZ European	10.6 (3.1)	0.61
	Maori	8.5 (0.7)	
	$\begin{array}{l} \text{Middle Eastern}\\ \text{Latin American}\\ \text{African} \end{array}\}$	13	
	Other	11.3 (0.6)	
Parent also has IBD	Yes	10.5 (2.1)	0.948
	No	10.6 (2.8)	
Other children have IBD	Yes	11 (2.8)	0.857
	No	10.6 (2.9)	
Time since diagnosis	<2 years	10.7 (2.9)	0.951
	>2 years	10.6 (2.9)	

### Previous Camp Attendance

The effect of previous attendance at Camp Purple education sessions was examined as a categorical variable for the number attended, which varied from zero to three times. This showed no significant difference between group IBD-KID2 score means (*p* 0.498), and when studied as a dichotomous variable of having attended or not, this was also non-significant (*p* 0.553).

### Participant Response Patterns

**Figure 1:**
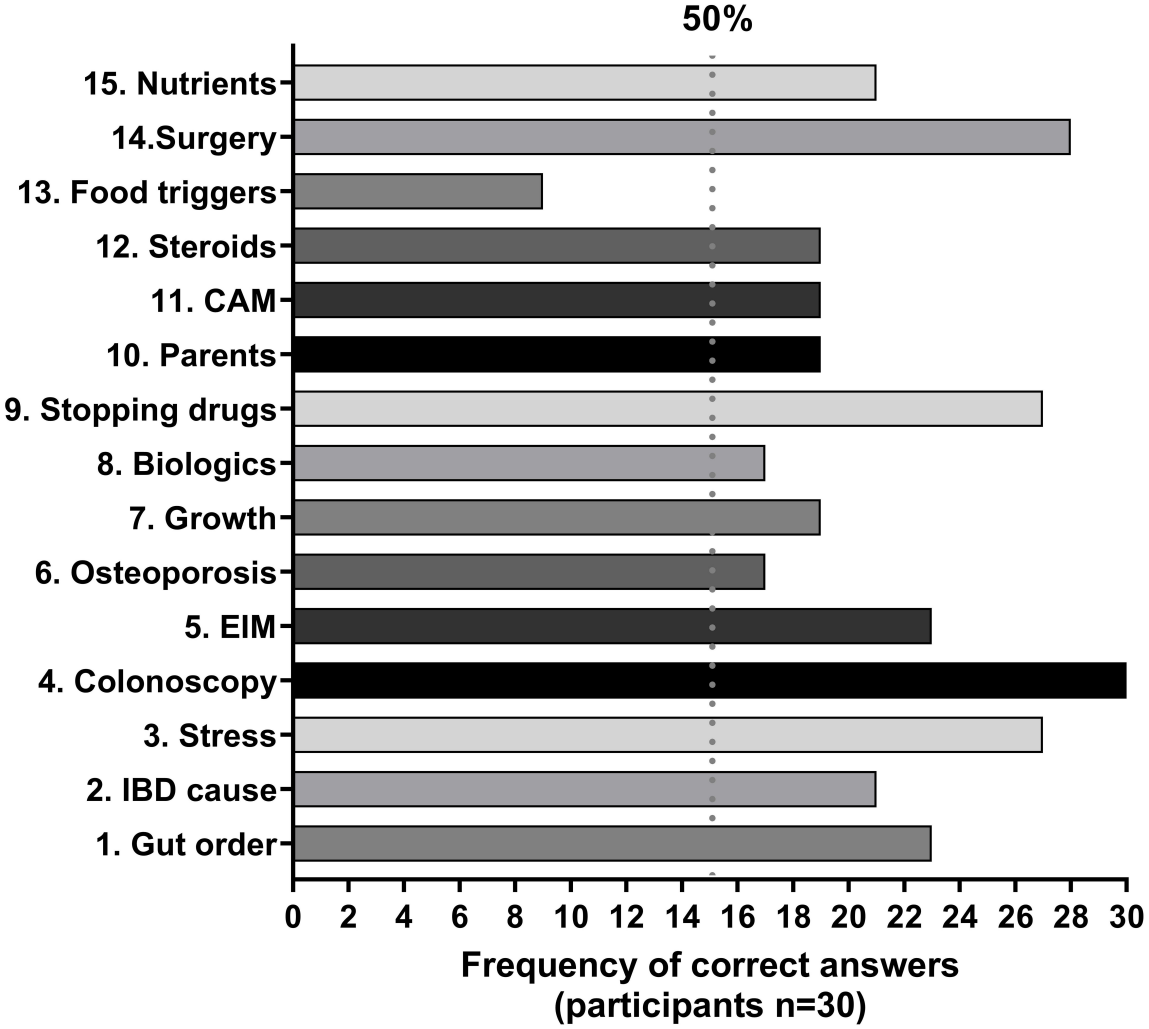
Frequency of correct responses given to IBD-KID2 items. The vertical line defines areas of good knowledge (>50% correct) and poor knowledge (<50% correct). CAM, complementary and alternative medicines; EIM, extra-intestinal manifestations.

The frequency of correct answers at baseline for each of the 15 items on IBD-KID2 was examined, showing that only one item was scored correctly by <50% of the cohort, thus indicating good overall knowledge levels (Figure 1).

### Repeat IBD-KID2 Comparisons

**Figure 2:**
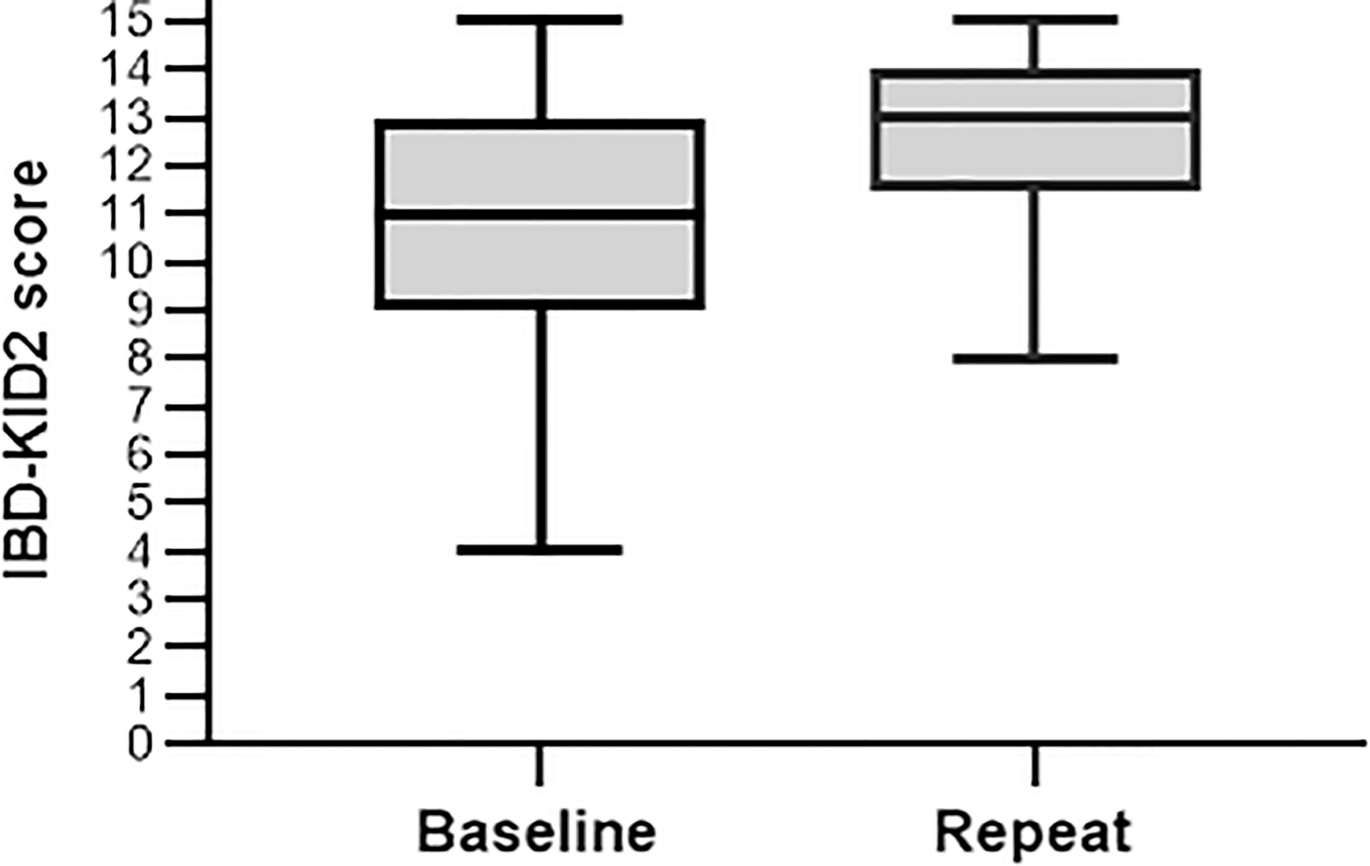
Change in IBD-KID2 scores from baseline to the post-education session repeat.

The 25 participants who completed IBD-KID2 at both time points represents an 83% response rate, with the mean time between assessments being 18.7 days (*SD* 3.9, range 14–25). There was no association with any independent variable for those parents who did not complete the second IBD-KID2 assessment. For participants with both data sets the mean score at baseline increased significantly from 10.9 (*SD* 2.8), to 12.6 (*SD* 2.0) following the education session (*p* < 0.005) (Figure 2). As can be seen (Figure 2), while the maximum score was achieved by some participants at both time points, the lowest score threshold improved by four points.

These score changes were examined at the individual level in the 25 parents, which showed that scores increased for 18 (72%), stayed the same for five (20%), and decreased by one point for two parents (8%). The initial baseline score was the only variable predictive of the magnitude of the longitudinal change (*R* 0.661, *p* < 0.005), all other independent variables were non-significant. The ICC of the participants repeat assessments was measured to determine how consistent their answers were when compared as a group. The ICC between the two time points was 0.8 (*p* < 0.005), depicting good internal consistency.

**Figure 3:**
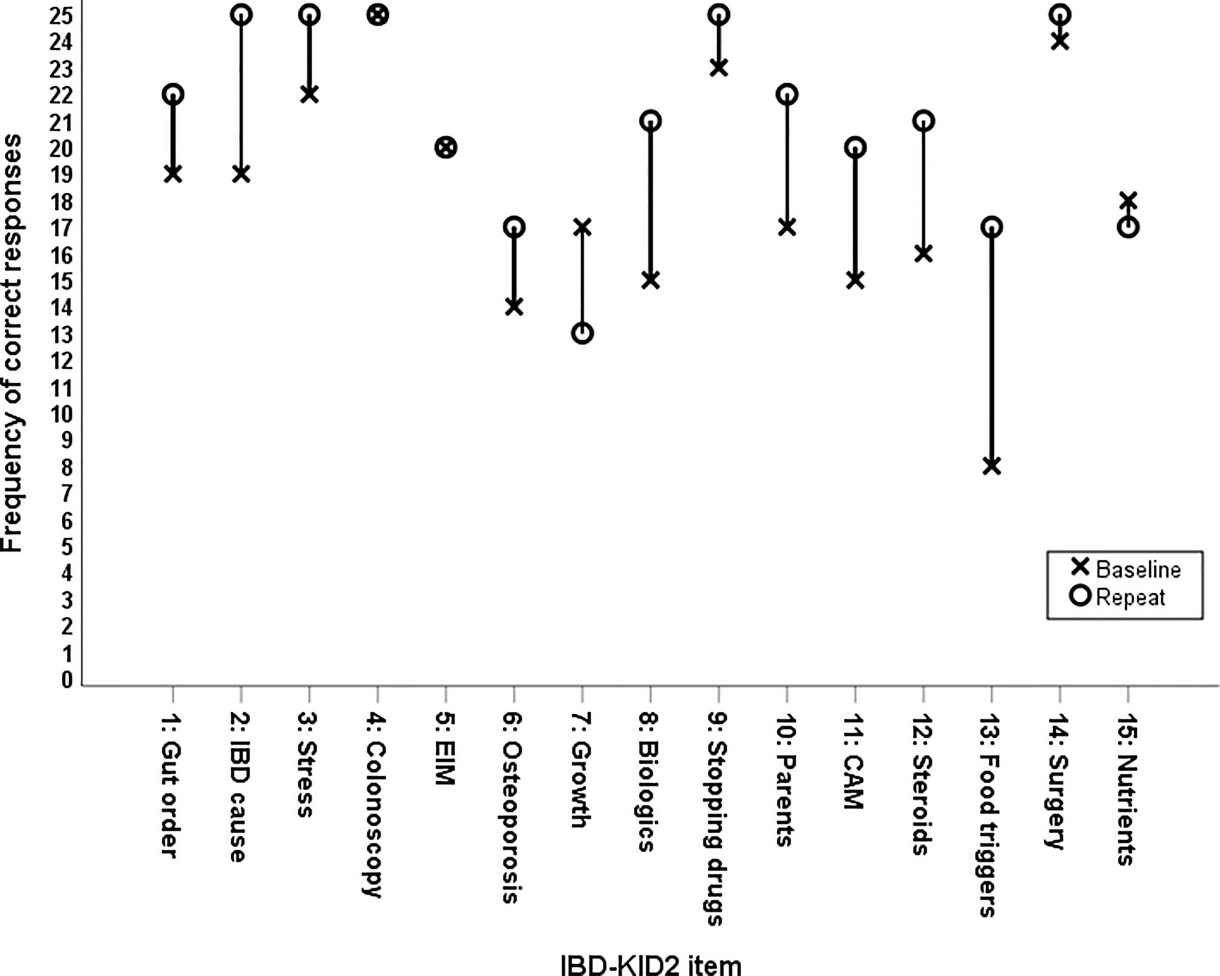
Change in frequency of correct responses between baseline and repeat administration for individual IBD-KID2 items.

The change in percentage of correct answers for each individual IBD-KID2 item was examined between the baseline and repeat administrations (Figure 3). In addition, the answer response “don't know” was used for 19.7% of answers at baseline, and 9.1% when completing the repeat IBD-KID2 (*p* < 0.005).

## Discussion

Opportunities for the clinical team to provide teaching in the hospital setting are limited by time and practical constraints. In this study a residential camp for children with IBD provided the ideal opportunity to present a parent education program. While evidence proving efficacy for interventions such as this is scarce, this study has shown that an education program can significantly improve parent disease-specific knowledge levels.

The level of parent education in this study had no significant effect on scores, as opposed to a previous study that showed parents with a post-secondary education had significantly higher knowledge levels (15). It should be acknowledged that this could represent differences in health literacy, not necessarily parental education level, however, this trait was not measured in either study.

A number of studies among children and adults with IBD have shown that membership of an IBD support group or society has produced significantly higher scores for respondents (6, 15–17). It is possible that our study population exhibited selection bias having been recruited at Camp Purple, but it is also likely that this group comprises those motivated to learn, as opposed to knowing more at baseline than those who chose not to attend.

The two lowest scoring items were regarding food triggers and osteoporosis, which were scored correctly by 33 and 58%, respectively. Similar knowledge gaps on these topics have been found in previous research among parents of children with IBD (15), but following the Camp Purple program correct scores for both items improved by up to 38%, thus highlighting the importance of education and teaching. Two IBD-KID2 items had fewer correct answers following the education session than at baseline, with the item regarding children's growth while in remission having the largest decrease. This topic has universally poor understanding among people with IBD, with correct answers to the corresponding item in an adult IBD knowledge assessment tool (CCKNOW) ranging from 13 to 50% (5, 16, 18, 19). Clarification was sought on whether the topic was covered in the education program, via personal communication with the coordinating pediatric gastroenterologist. It was confirmed that this was not discussed with parents but will be addressed in future years. The decline in correct answers for this item was, therefore, assigned to chance as opposed to misunderstanding.

The repeat administration of IBD-KID2 was performed to detect changes in IBD-KID2 scores, but the time-period was too short to measure whether the knowledge was retained long term. Previous work has shown adults with IBD retain knowledge following an education intervention for between 2 and 9 months (5, 6, 20). This study showed no significant difference in knowledge levels for those parents having attended previous Camp Purple education sessions. It may be argued that parents' exposure to different treatment modalities, or their child's disease course, may not have changed in that time-period and they may not have received recent teaching or educational materials. There should, therefore, be no expectation of higher knowledge levels but highlights that information should be reinforced during clinic visits regardless of disease duration and should be regarded as an ongoing endeavor.

### Limitations and Strengths

This study had no comparator control group that could determine whether parent knowledge levels may have increased without the intervention of the education program. However, the original IBD-KID2 validation study (14), and further work using IBD-KID2 (to be published) confirm that scores did not increase during a similar time-period for children with IBD, or their parents, when there was no intervention present. As discussed, the time period between each administration of IBD-KID2 in this study was insufficient to address whether knowledge was retained in the long-term and will be addressed in future work. The two administrations of IBD-KID2 were performed using different formats—the first on paper and the second online. It was shown during the original IBD-KID2 validation study (14) that this had no effect on children's scores, so it may be surmised that the same would be applicable to parents. The study had a good respondent return rate (83%) for the second online IBD-KID2 administration, possibly due to the convenience of not having to return paper versions.

The aim of this study was to establish whether a parent education session could improve parent's knowledge levels of IBD, and whether IBD-KID2 could detect the sensitivity to change pre and post intervention. The education program significantly improved parent's IBD-KID2 scores, thus addressing both aims. Performing this study at Camp Purple harnessed a rare opportunity as it allowed time for parents and clinicians to communicate and share knowledge in a setting that is conducive to clinical discussions, but does not have the constraints of children, time, and other clinical pressures that are present in an outpatient or inpatient setting. The benefits of residential camps for children and their parents are multifactorial, and this study adds to the growing literature by showing that parent education sessions can make a significant difference to the disease-specific knowledge of parents who have children with IBD.

## Data Availability Statement

The raw data supporting the conclusions of this article will be made available by the authors, without undue reservation.

## Ethics Statement

The studies involving human participants were reviewed and approved by University of Otago Human Ethics Committee (Health). Written informed consent to participate in this study was provided by the participants.

## Author Contributions

All authors made substantial contributions to the conception, design of the work, the acquisition, analysis, interpretation of data for the work, revised the work critically for important intellectual content, gave approval for the final draft to be published, and agree to be accountable for all aspects of the work in ensuring that questions related to the accuracy or integrity of any part of the work are appropriately investigated and resolved.

## Conflict of Interest

AD is Specialty Chief Editor of Frontiers in Pediatrics: Pediatric Gastroenterology, Hepatology, and Nutrition. The remaining authors declare that the research was conducted in the absence of any commercial or financial relationships that could be construed as a potential conflict of interest.
