# Supplementary material for: Assessment of Knowledge Levels Following an Education Program for Parents of Children With Inflammatory Bowel Disease
Source: Front Pediatr. 2020 Aug 12;8:475. doi: 10.3389/fped.2020.00475 (PMC7438864; doi:10.3389/fped.2020.00475)
Supplement: Supplementary file 1 [file Data_Sheet_1.PDF]

Supplemental digital content 1: Syllabus of Camp Purple parent education session

Day one delivered sessions on:

- An IBD overview: Paediatric Gastroenterologist
- Surgical aspects: Paediatric Surgeon
- Adolescent health: Paediatrician and Adolescent Health Specialist
- Coping with chronic illness: Paediatric Psychologist
- Crohn’s and Colitis New Zealand support group: Paediatric Gastroenterologist
- Question and answer session: Paediatric Gastroenterologists

Day two delivered sessions on:

- Old and new IBD therapies: Paediatric Gastroenterologist
- The nursing role in management of IBD: Paediatric IBD Nurse Specialist
- Nutrition and IBD: Paediatric IBD Specialist Dietitian
- The psychosocial impact of IBD: Paediatrician
- Epidemiology of IBD: Paediatric Gastroenterologist
- Discussions and questions: Paediatric Gastroenterologists, Nurse Specialist, Dietitian.
