# Supplementary material for: Assessment of Knowledge Levels Following an Education Program for Parents of Children With Inflammatory Bowel Disease
Source: Front Pediatr. 2020 Aug 12;8:475. doi: 10.3389/fped.2020.00475 (PMC7438864; doi:10.3389/fped.2020.00475)
Supplement: Supplementary file 2 [file Data_Sheet_2.PDF]

The
Inflammatory **B**owel **D**isease **K**nowledge **I**nventory **D**evice Version **2** (IBD – KID2)

Instructions:

★ This is not a test so don't worry about getting the answers wrong.

★ Please put a circle around the letter of one answer. If you don't know the answer put a circle around 'don't know'.

★ Please answer the questions by yourself. If you need someone to read the questions to you that is ok, but we need your answers.

★ Please don't study before answering the questions or ask for help to get the right answer.

★ Take as long as you like to finish IBD-KID2, but please finish the questions all at the same time. Don't leave half for later or another day.

★ Thank you for taking the time to fill this in.

1) From start to finish, the correct order of the gut is.:
   a) Mouth → stomach → oesophagus → large bowel → small bowel → anus
   b) Mouth → oesophagus → stomach → large bowel → small bowel → anus
   c) Mouth → oesophagus → stomach → small bowel → large bowel → anus
   d) Don't know

2) Doctors and scientists know what causes IBD.
   a) True
   b) False
   c) Don't know

3) Stress can trigger an IBD flare.
   a) True
   b) False
   c) Don't know

4) The reason you might have a colonoscopy of your large bowel is to:
   a) Look for disease
   b) Remove part of it
   c) Apply drugs inside it
   d) Don't know

5) IBD can affect other organs, not just the gut.
   a) True
   b) False
   c) Don't know

6) Which <u>one</u> fact about Osteoporosis (weak bones) is true?
   a) It doesn't affect males or young women
   b) If I drink plenty of milk I won't get it
   c) It can be caused by IBD
   d) Don't know

7) IBD that is in remission can slow down a young person's growth.
   a) True
   b) False
   c) Don't know

8) How do biologic drugs work?
   a) They reduce the chance of infections
   b) They block the chemicals or cells that cause inflammation
   c) They help the body absorb enough nutrients
   d) Don't know

9) If a person with IBD has had no symptoms for a few months they should stop taking their drugs.
   a) True
   b) False
   c) Don't know

10) If both parents have IBD their children will develop IBD.
   a) True
   b) False
   c) Don't know

11) Which <u>one</u> fact about complementary and alternative products is true? *(example – herbal drugs).*
   a) They may interact with prescribed drugs.
   b) They are natural so do not have side effects.
   c) They are all safe to use with prescribed drugs.
   d) Don't know

12) If you get side effects from taking steroids you should stop taking them at once.
   a) True
   b) False
   c) Don't know

13) Not eating some foods will stop your IBD getting worse. (*example - milk*).
   a) True
   b) False
   c) Don't know

14) Which <u>one</u> fact about IBD surgery is true?
   a) All people with IBD will need surgery
   b) Surgery is not helpful for people with IBD
   c) Surgery is helpful for some people with IBD
   d) Don't know

15) People with IBD can absorb all the nutrients they need if they eat the right foods.
   a) True
   b) False
   c) Don't know

Thank you for answering our questions! ☺
